# A framework for the promotion of ethical benefit sharing in health research

**DOI:** 10.1136/bmjgh-2021-008096

**Published:** 2022-02-10

**Authors:** Anja Bedeker, Michelle Nichols, Taryn Allie, Tsaone Tamuhla, Peter van Heusden, Olorunyomi Olorunsogbon, Nicki Tiffin, for the PHA4GE Ethics and Data-Sharing Working Group

**Affiliations:** 1 South African Medical Research Council Bioinformatics Unit, South African National Bioinformatics Institute, University of the Western Cape, Bellville, South Africa; 2 College of Nursing, Medical University of South Carolina, Charleston, South Carolina, USA; 3 Computational Biology Division, Integrative Biomedical Sciences, University of Cape Town, Cape Town, South Africa; 4 Department of Health promotion and Education, Faculty of Public Health, University of Ibadan, Ibadan, Nigeria

**Keywords:** public health

## Abstract

There is an increasing recognition of the importance of including benefit sharing in research programmes in order to ensure equitable and just distribution of the benefits arising from research. Whilst there are global efforts to promote benefit sharing when using non-human biological resources, benefit sharing plans and implementation do not yet feature prominently in research programmes, funding applications or requirements by ethics review boards. Whilst many research stakeholders may agree with the concept of benefit sharing, it can be difficult to operationalise benefit sharing within research programmes. We present a framework designed to assist with identifying benefit sharing opportunities in research programmes. The framework has two dimensions: the first represents microlevel, mesolevel and macrolevel stakeholders as defined using a socioecological model; and the second identifies nine different types of benefit sharing that might be achieved during a research programme. We provide an example matrix identifying different types of benefit sharing that might be undertaken during genomics research, and present a case study evaluating benefit sharing in Africa during the SARS-CoV-2 pandemic. This framework, with examples, is intended as a practical tool to assist research stakeholders with identifying opportunities for benefit sharing, and inculcating intentional benefit sharing in their research programmes from inception.

**Summary box**

- There is an increasing recognition of the need to include benefit sharing in research programmes, but translation and the practical implementation of benefit sharing is slow.
- It can be difficult for researchers to operationalise benefit sharing in their programmes without practical guidance about the different forms benefit sharing might take.
- We have developed a benefit sharing framework with two dimensions: we use a socioecological model to identify microlevel, mesolevel and macrolevel stakeholders for the first dimension, and for the second dimension we define nine different types of benefit sharing that can be implemented.
- We provide a matrix showing the application of the framework to identify opportunities for benefit sharing in genomic research undertaken in Africa, and present a case study of benefit sharing arising from the SARS-CoV-2 pandemic in Africa.
- The framework is designed to provide a practical tool for researchers, funders, ethics review boards and other stakeholders aiming to identify benefit sharing opportunities in research programmes.

## Introduction

**Figure 1:**
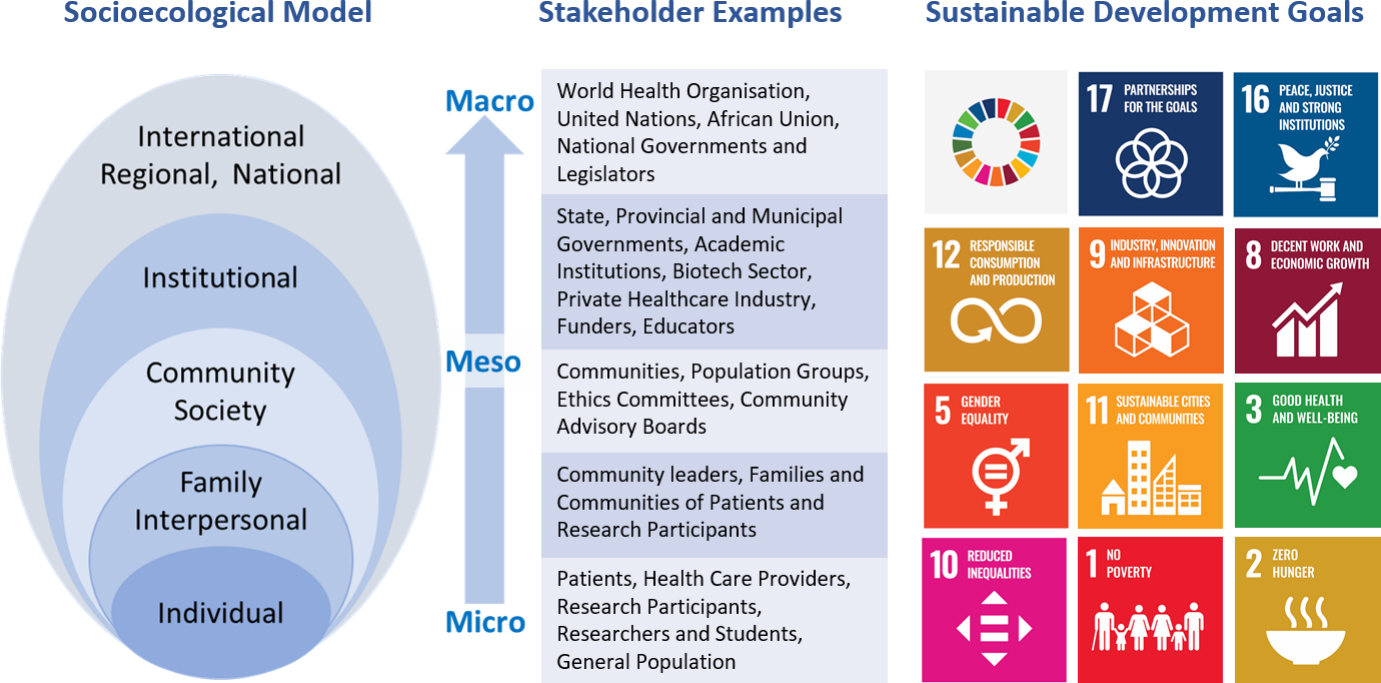
Outline of the socioecological model, with examples of stakeholders and corresponding Sustainable Development Goals.

 Ethical management of the data and biospecimen ecosystem for health research is underpinned by a fundamental principle of balancing risks and benefits in the pursuit of respectful, equitable and meaningful research to benefit humanity (reviewed in^1^). While health research directly contributes to achieving the United Nations’ Sustainable Development Goal (SDG) 3, Good Health and Well-being, there is scope for the research ecosystem to provide many more benefits that can contribute to many more SDGs including reducing inequalities, contributing to socioeconomic goals and supporting sound policy-making institutions and partnerships. In figure 1, we provide examples of SDGs to which health research could contribute, such as capacity development to improve stakeholder partnerships and support institutions (SDGs 16, 17), optimising efficient and responsible use of resources for healthcare (SDGs 11, 12), stimulating economic activity through development of the health and biotech sector (SDGs 8, 9) and ensuring a healthy, employed workforce with reduced risk of poverty and hunger (SDGs 1,2, 3, 10). Health research stakeholders can also actively choose to reduce inequality and promote gender equality during their activities (SDGs 5, 10). For every health research study undertaken, in order to uphold practices that are equitable and just it is therefore important to interrogate who will take which risks, what are the benefits and to whom will those benefits accrue. Here, we discuss benefit sharing in terms of the actions taken towards ensuring that various benefits of research are shared with a wide range of stakeholders in a way that is equitable and just.

Discourse about ensuring the fair distribution of research benefits has been ongoing for decades, emerging initially from clinical research involving human participants and subsequently extending to health genomics research.^2^,^5^ For non-human biospecimens, in relation to bioprospecting, the Nagoya Protocol aims to inculcate benefit sharing alongside material transfer agreements, but human biological resources and digital sequence information are not currently included.^6^,^8^ In 2016, the Council for International Organizations of Medical Sciences updated their research ethics guidelines to discuss benefit sharing for medical research and to advocate for negotiated benefit sharing agreements, but failed to explore the impact of inequity on the negotiating position of stakeholders in the global South.^9^ Benefit sharing can take many forms: the most tangible is often the sharing of financial benefits from research with research participants, which occurs at the level of individuals; but there can be many other types of benefits which may have a wider scope beyond the individuals who directly participate in research. These might include developing capacity and infrastructure, increasing skills and knowledge, and providing wider career opportunities and exposure to research stakeholders. Better access to healthcare and/or improved health facilities and advances in the types of healthcare available are all benefits that can reach the wider populations where research has taken place (table 1).

**Table 1:** Summary of the elements of the two-dimensional benefit sharing framework

**Dimension 1: StakeholdersMacrolevel stakeholders**These stakeholders generally make decisions and provide services at a national or higher level, and include global and regional organisations, national organisations, governments, policy makers, regulatory bodies and organisations, legislators and public health officials. For example, The WHO, the African Union or the Government of South Africa.**Mesolevel stakeholders**These stakeholders may impact provincial, state, municipal or institutional level. They may include some larger community groups. Examples include Academic institutions, ethics review boards, particular population groups, provincial governments, provincial health services, funders, educators, biotech or private health service companies, institutions and facilities.**Microlevel stakeholders**These are individuals, families or small community groups who operate at a personal or interpersonal level. For example, Members of the general population, research participants and their families, researchers and students, healthcare providers, patients, community leaders and community advisory boards.	**Dimension 2: Benefit sharing categoriesFinancial:** Direct monetary gain by stakeholders.**Health and well-being:** Improved individual and/or population health and well-being for stakeholders.**Infrastructure:** Built or logistical infrastructure that benefits stakeholders.**Equipment:** Specialised equipment used by stakeholders to conduct their work.**Skills capacity:** Learnt specialised skills to undertake tasks and conduct work.**Knowledge:** Specialist knowledge that improves how stakeholders solve problems, undertake tasks and conduct work.**Services capacity:** Capacity for stakeholders to provide certain services to the public and general population.**Career development:** Opportunities for stakeholders to establish employment security and/or progress in their careers.**Attribution and recognition:** Appropriate acknowledgement and advertising of inputs and contributions from stakeholders.

To date, individual research participants in the global South rarely experience benefits from the research in which they participate.^10^ Recent Ebola virus outbreaks in West Africa and the current global SARS-CoV2 pandemic have starkly highlighted this in Africa: Samples from Ebola patients appropriated by teams from the global North were used for commercial development without consent or benefit sharing agreements with countries, communities or patients of origin^6 11^; and COVID-19 vaccine scarcity and delays in access for adults in Africa persist in the face of booster shots for adults and child vaccination in the global North despite willing African participation in COVID-19 vaccine research and trials.^12^,^14^ Allegations of misuse of African DNA for unconsented commercial applications with unshared benefits persist.^15^ Ethics dumping, whereby foreign researchers undertake research in the global South under lower ethical standards than would be tolerated in their home countries, is still common^10^; and predatory, or ‘helicopter’ research in which foreign researchers fly in to collect samples, often treating local collaborators as sample collectors and excluding them from participating further in the research process, is still practiced across the global South.^16^ Experiences of inequitable research practices often underlie distrust in research by individual participants through to communities (eg, Havasupai,^17^ San^10^ and First Nations Australian^18^ communities), institutions and at national and regional levels.^19^ Similarly, researchers in the global South are also often distrustful of collaborations with global North institutions, although they may need to engage in such alliances to access research funding.^20^ Inequitable and/or neocolonial practices and power imbalances still underpin many ongoing research interactions between the global North and global South.^21^,^24^

In an effort to improve such practices, various communities and populations have set up requirements and guidelines for researchers who wish to engage with them, for example, the Te Mata Ira Guidelines for Genomic Research with Māori in New Zealand,^25^ The San Code of Research Ethics,^26 27^ and recommendations for research engagement with North American Indigenous groups.^28^ Regional guidelines increasingly enshrine more equitable research that includes benefit sharing, such as the African Academy of Sciences Data and Biospecimen Governance Guidelines.^29^

Onward data sharing has achieved global attention, and is normally a requirement to receive funding and publish research while benefit sharing mostly remains an afterthought: funders, journals and ethics review boards require data-sharing statements without asking for benefit sharing statements or plans; data sharing is often enforced, even where inequality might compromise the interests of those collecting the resources,^30^ and yet benefit sharing does not receive even a fraction of this attention. Community opinions on providing benefits and payments to participants commonly focus on financial benefits and elaborate on fairness of compensation, particularly for participants with very limited resources.^31^,^33^ In order for benefit sharing to be fair, considering proportionality is also important in terms of the benefits accruing to meso and macro-level stakeholders, who may take smaller relative risks compared with exposure to direct personal risk experienced by individual research participants.^9^

What can shift this unfortunate status quo? Increasingly, researchers in the global North are becoming sensitised to global inequities in research practices and showing increased willingness to engage with benefit sharing concepts.^34^ Currently, this is largely abstract engagement with limited translation of the concept of ‘benefit sharing’ into a clear strategy to encourage more equitable practices. The process of operationalising benefit sharing requires involving a wide range of stakeholders, beyond just researchers and participants, in normalising and inculcating benefit sharing in research undertaken in the global South. It requires advocacy with ethics review boards, peer-reviewed journals and research funders as well as ongoing consultation with participants and their communities about what might be acceptable benefits, beyond remuneration, in return for research participation.

## A framework to guide operationalising benefit sharing in research

Taking the next step towards routine implementation of benefit sharing requires practical guidance on how benefit sharing might be implemented. Towards this goal, we present a benefit sharing framework that provides researchers with a practical resource to help identify ways to share benefits during health research (table 1). The development of this framework has been informed by the shared experiences of the authors undertaking research in the global South and participating in various health research consortia and networks with strong ethics components as well as capacity development programmes.

The framework is defined by two dimensions, namely Categories of Benefits, and Stakeholders. It is intended to provide an overarching structure to assist with identifying benefit–sharing opportunities, and could be applied across a variety of different contexts and health research domains informed by domain-specific experience and knowledge.

### Categories of benefits

We have defined six different categories of benefits that move beyond the most commonly recognised form of benefit sharing based on financial returns such as financial return from translated pharmaceutical products to financial payments to research participants.^35^ In addition to financial benefit sharing, we characterise more intangible types of benefits in categories of health and well-being, infrastructure, equipment, skills capacity, knowledge, services capacity and career development, attribution and recognition.

### Stakeholders

In order to have an inclusive and holistic approach to stakeholders and the different types of benefits they might experience, we have adopted a modified socioecological model to identify microlevel stakeholders at the individual and community level, mesolevel stakeholders such as institutions and organisations, and macrolevel stakeholders such as governments and regional or international organisations. We provide an overview of the types of benefit sharing we have identified at these different levels for the defined benefit sharing categories (figure 1), recognising that stakeholders will potentially both receive and distribute different types of benefits in relationships with other stakeholders at various stages in the research life cycle: the framework structure allows for this fluidity, as distinct benefit sharing activities can be individually characterised where the elements of the two dimensions intersect.

Microlevel benefit sharing: To date, perspectives on benefit sharing have often focused at the micro level on research participants and patients, their families and immediate communities where research is taking place.^3 7 36^ Benefit sharing at this level might be financial, by remuneration of participants for their time, inconvenience or discomfort, or by the provision of vouchers to buy groceries or mobile phone airtime. Participants might perceive additional health and well-being benefits that include convenient or free access to healthcare, or privileged access to specialist healthcare not normally available to them. In these circumstances, participants might also confuse research-related access to healthcare with provision of standard healthcare services, not realising that they are receiving such benefits only due to participation in a research programme. This highlights the importance of providing explicit participant information about the research programme, with clear descriptions of potential risks and associated benefits and the voluntary nature of participation.^37^,^39^ Communities and members of the public might also benefit from improved infrastructure and improved health services available, for example, upgrades to public health clinics and facilities and equipment such as diagnostic machinery that are brought in for research purposes and then continue to be used after study completion. Individuals in the community may gain new knowledge through exposure to the research ecosystem and by participating in ongoing studies, and gain skills and local recognition through participation in community advisory boards.

Research programmes can also provide benefits to postgraduate students and researchers, who might directly receive stipends and salaries to support their training and employment and who can access additional training, mentorship and learnerships to increase their skills and knowledge. They may also be able to leverage improved infrastructure and capacity to further their own work as well as accessing onward career and educational opportunities through an expanded research network and opportunities to present or showcase their contributions.

Mesolevel benefit sharing: At the mesolevel, we have identified provincial, regional and municipal stakeholders such as organisations or governments who may be responsible for delivery of health, education and other services in their region; populations and communities; universities and academic institutions and healthcare facilities. Other mesolevel benefit sharing stakeholders include funding organisations that represent both governmental and philanthropic funding sources. Stakeholders in the biotech sector include biotechnology and service delivery companies, and commercial health services.

At the institutional level, research programmes may focus on building long-term infrastructural capacity through refurbishing facilities and investing in research equipment. Skills development can also permeate to an institutional level through the training of a new cohort of skilled local trainers who will disseminate skills into the future. As an example, the Human Heredity and Health in Africa (H3Africa) programme (www.H3Africa.org) is an African-wide Consortium funded by the National Institutes of Health (USA) and the Wellcome Trust (UK) to undertake collaborative health genomics research on the Continent. The H3Africa programme inculcated capacity building and training as key programme deliverables^40 41^ to promote onward sustainability of health genomics research in Africa, with regional investments in biobanking infrastructure as well as a large bioinformatics training programme across the Continent.^26^ In addition, institutions benefit when research funding financially supports staff who rely on ‘soft-funded’ salary support. Often such funding as well as percentage awards from grants for institutional overheads is essential to keep tertiary research institutions viable where government funding is insufficient, and helps institutions to increase their profile, reputation and consequently their sustainability.

With respectful and culturally appropriate community engagement in place, communities and particular population groups might also benefit from research programmes through enhanced science and research competency, increased understanding of their ethical, legal and human rights, and opportunities for increased scientific citizenship. The Summer Internship for Indigenous Peoples in Genomics Consortium provides an admirable example of community-level benefit sharing through training and scientific citizenship in Canada, the USA, Australia and Aotearoa/New Zealand.^28 42^

Macrolevel benefit sharing: Policy and legislation established at national, regional and global levels requires a strong body of evidence to drive decision making. Focused and topical research programmes can return benefits at this level through the provision of relevant and reliable research. While research is often published in peer-reviewed journals, a benefit sharing component to be added is the compilation and distribution of executive summaries to relevant national and international policy-makers. Currently, the translation of cutting edge research to practice and policy is slow, with a concomitant lag in the return of benefits of research to global populations through improved policy and better implementation.^43^,^46^ Concerted collaborative global efforts during the COVID-19 pandemic have illustrated how the research-to-practice gap might be more efficiently reduced in the future.^47^

## Exemplar matrix and case study

To illustrate the application of the framework for benefit sharing in health research, we present an applied example by populating a matrix that identifies a variety of stakeholders who could benefit from health genomics research in the global South, and describe different types of benefits that they might realise using the framework benefit categories (online supplemental data file). In the matrix, we provide some practical examples of how benefit sharing could be implemented to benefit microlevel, mesolevel and macrolevel stakeholders using the example of genomics health research, recognising that the matrix may not identify all stakeholders and opportunities for benefit sharing, but instead presents an evolving set of scenarios to assist and enable practical application of benefit sharing while conducting health genomics programmes undertaken in the global South. It is our intention to illustrate how researchers might use this framework at the planning stages of their own programmes to identify important microlevel, mesolevel and macrolevel stakeholders, and consider for each the benefits that might be created and shared under each category. It may also assist funders and ethics review committees in how they operationalise an intention to inculcate benefit sharing in research practices, and how they might assess benefit sharing plans in ongoing research.

In addition, we present a case study using the framework as a guide to assess benefit sharing at microlevel, mesolevel and macrolevels in COVID-19 research within Africa during the current pandemic, discussing successes, missed and future opportunities for benefit sharing (box 1).

Box 1 **Case study: distributed benefits from SARS-CoV-2 genomics research in Africa**

The SARS-CoV-2 pandemic emerged in Africa in February/March 2020, brought in by international travellers and subsequently spread between countries by both land and air travel. African countries have experienced stressed health services, inadequate public health infrastructure and enormous social, economic, health and mortality burdens for African populations.

Microlevel benefits: In September 2021, as the third pandemic wave was slowly subsiding in South Africa, a coalition of authors published a research article describing genomic surveillance in Africa during the prior year and the phylogeny of 8746 publicly available genomes.^48^ Researchers involved in this study had new opportunities open up to them to further this research in the interests of public health and were involved in new and wider collaborations for COVID-19 research.^49^ In addition, many academic researchers and postgraduate students were able to contribute to the public health effort through laboratory-based contributions, training and through data analysis expertise.^50^,^52^ For COVID-19 patients and the general public, expedited SARS-CoV-2 public health, biomedical and genomic research was able to rapidly feed evidence-based approaches to containing the pandemic: very quickly there were epidemiological and modelling studies that informed scenario planning and helped to identify modes of transmission, leading to public health advice about social distancing and mask wearing, incubation and isolation periods for viral infection.^53 54^ Existing PCR and qPCR technologies were quickly employed to develop and test rapid diagnosis tools; potential therapies were rapidly tested in clinical trials, and the development of effective vaccines was undertaken at an unprecedented rate.^55 56^ Members of the public in some countries were also able to participate in trials for vaccines that were subsequently shown to be effective. All of these developments also enhanced translational collaboration between the public health service delivery sector, academic research and the biotech sector in order to contain the pandemic, protect individuals from infection and preserve the health of all members of the global population. Identifying new variants as they appear in different populations has also helped to drive evidence-based policy and interventions to protect members of the public and ensure diagnostic tests and vaccines remain effective.^57 58^

Mesolevel benefits: While existing infrastructure allowed rapid sequencing of the pathogen as cases emerged initially in Nigeria, South Africa and Egypt, and other African countries, capacity limitations such as lack of equipment, infrastructure and technical skills have all slowed down the translation of genomics research into health policy. In response to the pandemic, funding has been provided to various institutions and collaborative groups, both in academic and service delivery roles, who have been able to build on existing programmes, and establish and fund new collaborations.^59^,^62^ Collaborations between public and private sector organisations were also ramped up, with next generation sequencing companies Illumina and Nanopore partnering with Africa Centre for Disease Control CDC), and increased interactions with courier facilities to ensure effective logistical coordination for sample transport. Governance guidelines were also rapidly developed to promote equitable and ethical use of samples and data.^63 64^

Macrolevel benefits: At the policy level, the WHO is strengthening their support of the African pandemic response, with a particular focus on vaccine accessibility and equity.^65^ The Africa CDC (www.africacdc.org) has also received substantial funding support to assist with their pandemic response, and has partnered with the United Nations Development Programme (UNDP) and African Union to develop enhanced capacity for pooled procurement of diagnostic and other medical resources, surveillance and reporting, and to support regional organisations and partners in their pandemic response.^66^ The collaborative efforts identifying new and potentially more virulent variants using genomic sequencing have also informed the Variant of Concern list published by the WHO for global reference^67^, with the detection of the Beta variant in South Africa leading to shifts in vaccine and travel policies.^68^

Missed opportunities and future directions: In the scramble to develop a medical response to COVID-19, opportunities for wider community engagement were missed, along with opportunities to share benefits such as increased accessible knowledge, increased scientific citizenship and population health literacy for the general public, and community participation in developing a COVID-19 response that can inspire public buy-in and support. In the absence of an inclusive approach and inculcated benefit sharing, ‘fake news’ and conspiracy theories have flourished, vaccine hesitancy has proved one of the largest obstacles to curbing the pandemic, and the social compact between health scientists and the general public is at an all-time low.^69^,^72^

Learning from successes and identifying missed opportunities can inform strategic policy around scientific research to support public health. The pandemic has highlighted the need to address global health inequity which has been harshly exposed through inequitable SARS-CoV-2 vaccine access despite the participation of the general population in global South countries in extensive vaccine trials.^73 74^ Better models for benefit sharing, developed through an intentional approach that includes community involvement in an equitable relationship, can start to address some of these gaps in global health equity, scientific citizenship and a more trusting relationship between the health science sector and the general public. This can be achieved in part by inculcating a benefit sharing mindset in the research ecosystem, and assisting researchers in operationalising benefit sharing from the earliest planning stages of all research programmes.

## Conclusion

In identifying different types of benefits that might be shared, it is essential to consider that individuals and their communities may have a wide range of motivations for participating in research, and may have different priorities with regards to the type of benefits anticipated as well as who will accrue them. Research programmes conducted in the global South, especially those funded externally and by the global North, would be well advised to engage meaningfully with each community involved in health research to understand where some of these local and individual priorities might fall, recognising the autonomy and self-determination of global South research participants in defining the terms of their preferred benefit sharing arrangements. Global North researchers will do well to consciously move from doing benefit sharing at global South participants towards building benefit sharing programmes with those stakeholders.

We have presented here a framework that can be used as a practical tool to identify ways of benefit sharing that extend beyond financial benefits provided directly to participants and patients at the point of interaction in research programmes, and rather recognise a wider range of potentially less-tangible benefits that can be extended to a variety of stakeholders. Many of these benefits may already be reaching stakeholders through more ad hoc routes in ongoing research programmes, but we believe that adopting an intentional and purposeful approach to identifying and enabling this kind of benefit sharing can help to operationalise existing intentions to undertake benefit sharing during research. When designing a new research programme, the researchers can use this framework to construct a benefit sharing matrix for their project—where needed, referencing the example matrix for health genomics research provided here as supplementary data, to assist in imagining practical ways to benefit share. In this example, we have used the health research benefit sharing framework to populate a matrix with stakeholders and types of benefits that could be shared in health genomics research in Africa. For these stakeholders we have identified types of benefits that might result from health genomics research, providing examples where those are already available.

In addition, scientific journals, funders and ethics review boards can assist with inculcating benefit sharing in research programmes through requesting benefit sharing plans and statements for all research projects, and this framework can similarly inform approaches to requiring and reviewing benefit sharing plans alongside data-sharing plans in research proposals and published manuscripts.

In developing our case study (box 1), we fully recognise the critical need for expediency in assessing transmission modes for the SARS-CoV-2 virus, and in developing and implementing vaccinations to curtail the severity and spread of the virus. As such, soliciting community input at all stages of the epidemiological, modelling, vaccine development and even translational studies for vaccine distribution lacked optimal public and community-specific input. Further delay in processes to ensure wide-spread input on community perspectives and preferences could have resulted in increased morbidity and mortality. However, in planning for future public and global health crises and to promote full consideration of community preferences and to optimise benefit sharing, investigators, providers, funders and policy-makers should foster such partners early on so these relationships are well established before there is a pressing need. The case study illustrates that a variety of benefits—which were identified by considering the types of benefit sharing in the framework—could be shared with microlevel, mesolevel and macrolevel stakeholders at many touch points during the COVID-19 pandemic, and that inculcating benefit sharing in everyday research practice could ensure that benefit sharing opportunities are utilised even in times of crisis. It also shows that the failure to consciously share benefits of scientific progress with the general public, at the micro level, has impeded public support for evidence based, scientific responses even while benefits have accrued to meso and macro stakeholders.

Had we already had true community-engaged partnerships before the start of the COVID-19 pandemic, the scientific community would have had greater insight into the perspectives of diverse populations, which may have resulted in decreased vaccine hesitancy. Taking a prospective approach now that considers ongoing benefit sharing, including bidirectional knowledge and skills sharing, can ensure that the voices of global populations are heard regarding scientific advancements that directly and indirectly affect them, and that scientific teams are well poised to ensure promotion of equitable benefit sharing for all, especially during times of public and global health crises.

This benefit sharing framework is a work in progress, and we anticipate that it will continue to evolve with input from interested parties and as benefit sharing becomes inculcated in health research, especially when it is undertaken in the global South. We invite and welcome comments and conversations towards this evolution from all interested parties.

## Supplementary material

10.1136/bmjgh-2021-008096 online supplemental file 1

## Data Availability

No data were used in this study.
