# Supplementary material for: A framework for the promotion of ethical benefit sharing in health research
Source: BMJ Glob Health. 2022 Feb 10;7(2):e008096. doi: 10.1136/bmjgh-2021-008096 (PMC8845198; doi:10.1136/bmjgh-2021-008096)
Supplement: online supplemental file 1 [file bmjgh-2021-008096supp001.pdf]

<u>Supplementary Data File:</u> Matrix showing application of the benefit sharing framework to potential benefits from genomics research in Africa

	Types of benefits:	Financial	Health and Wellbeing	Infrastructure	Equipment	Skills capacity	Services capacity	Career development, atribution/recognition	Knowledge
**Sociecological model**	**Stakeholders**								
**Macro**									
Policy	Global organisations e.g. WHO, UN	Financial support from member countries.	eg. WHO recommendations, operational response in COVID-19 driven by research as it is published. Evidenced-based responsiveness to global challenges - can be provided through accessible reports and policy briefs.	Highlight and focus where inputs are required, leverage research to appeal for funding for progammes e.g. COVAX, GAVI at WHO.			Knowledge, treatment, medication, etc. increases organisational ability to respond better and more rapidly to a regional or global health crisis.	Relevance of role of WHO highlighted through evidence-based and surveillance research.	Having access to up-to-date real data can aid in decision making processes e.g. when approving certain tests/methods for screening and diagnostics purposes, such as the decision to use the molecular based line probe assay in TB drug susceptibilty testing; can also enhance organisational ability to respond timeously to emerging health issues e.g SARS-CoV-2 currently.
Policy	Regional organisations e.g. AU, EU	Access to resources to tackle new public health threats. e.g. the current epidemiologic shift in sub-Saharan Africa, where we are seeing an increasing burden of non-communicable diseases.					Knowledge, treatment, medication, etc. increases the ability of organisations to coordinate a better and more rapid response to regional health crises.	Organisation's reputation is enhanced through participation in successful projects.	
Policy	National organisations, governments	Cheaper and more effective treament strategies and effective disease prevention programmes based on knowledge gained from research can allow for less government expenditure.		Benefit from new facilities being built or old facilites getting upgraded to better support genomic research.	New resources developed for research that may be used at a national level. e.g. High Performance Computing Facilities, Cloud data storage facilities, Biobanks.		Knowledge, treatment, medication, new/upgraded infrastructure and equipment etc. increases organisational ability to respond better and more rapidly to health crisis and offer more services.	Reputation and trust in government is enhanced through successful projects and interventions.	Have up-to-date data that is country- and population-specific can be used for evidence-based decision making.
Policy	Provincial organisations, local/city government				New equipment used for research and future health care delivery e.g. thermal cyclers for pathogen diagnostics.				
Policy	Policy makers								Have up-to-date data can aid in evidence-based decision making. Executive summaries of research findings can provide a synopsis of relevant new information that might inform policy.
Regulatory (guidance & policy, enforcement)	Regulatory bodies and organisations					Research programmes may contribute guidance and inform policy addressing benefit sharing through pilot benefit-sharing programmes (Lairumbi et al 2011, https://www.ncbi.nlm.nih.gov/pmc/articles/PMC3200159/pdf/1472-6939-12-20.pdf)	New challenges elicit new thinking horizons and help to broaden the scope of services to be offered, e.g. Testing services that were rapidly developed during the COVID-19 pandemic.		
Regulatory? (guidance & policy, enforcement)	Legislators								
Governmental	National public health officials	Research funding may financially support public health officials through salary support or providing a budget to help to achieve desired outcomes at work.	Access to resources that can be provided to healthcare clients on behalf of national public health offices, eg. access to affordable new medications, vaccines.	National Public Health organisations may have infrastructural investment through collaboration with researchers, such as public health facilities being developed or equipped.	Public health offices may benefit from the installation of equipment, including computational and data-storage equipment, during research programmes.	Public health officials may be able to access training, workshops and conferences through research collaborations, thus learning new skills and enhancing knowledge.		Collaborations with research programmes may elevate professional profiles and enhance CVs.	Reports provided of findings with public health relevance highlighted, to assist public health officials in with service delivery.
**Meso**									
Provincial, state or municipal	Provincial-level delivery of care		Rapid response to arising issues e.g. pathogen sequencing during the COVID-19 pandemic						Better decision making, arising from improved evidence resources
Institutional	Health care institutions			Research programmes may invest in health care facility infrastructure whilst conducting facility-based research	Research programmes may invest in medical equipment, which remains at the facility, in order to conduct research	Training on the use of certain machines/procedures, and possible introduction of skills in new diagnostic methodology; transfer of knowledge from research clinicians and staff to facility staff.	Information available through gene analysis could help improve service rendered, such as a genetics counselling service, as well as the development of more effective approaches to diagnosing and treating disease. Examples include genetic tests for newly identified monogenic diseases, as well as panels of variants employed for genetic testing for polygenic diseases or those with many causative genes.		
Institutional	Academic institutions	Funding of departments, staff and students		Upgrading of facilities, for exampe Stellenbosch University installed a state of the art biorepository unit; H3Africa has a specific capacity building agenda, reference: Dauda et al: https://pubmed.ncbi.nlm.nih.gov/29446211/	Access to new technology as and when it becomes available; perhaps getting new equipment that can be piloted, for exploratory analyses or for training.	Could attract a certain caliber of academics and students, training on the use of certain machines/procedure eg bioinformatics (e.g. H3ABioNet training in Africa)	Curriculum upgrade to accomodate new knowledge available	Could become a center of excellence if they piooner groundbreaking research; allow for opportunities to collaborate on an international scale	
Community	Communities - groups of people with something in common (identity, interests, beliefs, experiences, geographical/lived environment)		Targetted interventions if a community is disproportionately affected by a health condition	Resources (physical and human via clinical personnel with training and expertise) available; https://bmcmedethics.biomedcentral.com/articles/10.1186/1472-6939-15-90		Improve research competency through continous engagement with researchers	increase understanding of ethical and unethical research, protective legislation and their rights		Better understanding of the drivers of certain health issues which could help in prevention, management and / or treatement
Community	Population groups - group of people with shared ancestry		development of personalised medicine						
Institutional	Funders		Investements made toward health discoveries; ability to teams to compete for intramural funding			Cadre of scientific experts to partner with for application review panels/study sections. Internal expertise via project directors, program officers		Recognition for contributions (funding commitments) made to invest in scientific advancements. Inclusion of funding support in dissemination efforts. Publication opportunities with scientific teams.	Direct, larger scale insight and knowledge into the state of genomic science, expertise withihn the field, and easily identifiable gaps/priorities
Institutional	Institutional Ethics Review Boards	Income generated from fee for reviews				Opportunities for training on ELSI of genomics study	Ability to provide consultative services on grant applications and to ethics boards on human subject protections in genomic research and on benefit sharing.	Develop expertise in genomic research studies and benefit sharing. Serve as subject matter experts. Can contribute toward accreditation requirements of ethics committees.	Develop (individual and collective) expertise in genomic science and benefit sharing.

Institutional	Educators	Funding to undertake research projects			Get new equipment that aids their educational activities, extends the scope of what can be taught	Be involved in ground breaking research; training opportunities/workshops made available through research efforts	Grant writing services/collaborations made possible through funding opportunities. Teaching assistants/fellows available for educational program needs (lecture prep, undergraduate section teaching, grading assistance) based on stipends paid to students via grants.	Possible career advancement through publications of research and supervision of graduate students (Masters, PhD and Post_Docs); Ability to attract students and collaborators	Highly sought after intellectual expertise at institutions with strong research funding portfolios
Institutional ?	Biotech companies/sector incl private health services	Income from scientific advancements or services utilized	Products are developed					Recognition for project based involvement	
**Micro**									
Interpersonal	Community leaders	compensation for community participation			Equipment may be provided to support community activities	Leadership skills may be enhanced	Acess to accurate information that is beneficial to the community eg interview of community leaders may increases their understanding of a subject matter.	Having influence over community activities, resources and outcomes	Understanding of research, processes, ethics and outcomes, as well as better understanding of rights and protections and how to uphold these. More knowledge about advocacy for rights and protections
Interpersonal	Community advisory boards	Compensation for community participation			Equipment to support board meetings may be provided e.g. furnishing a meeting room	Leadership and advocacy skills may be enhanced through engagement with researchers; may be involved with data collection or participant recruitment within the community, depending on the focus of the research.		Possibility of being included in articles/publication/newsletters as authors. Respect from community members. May lead to opportunities for expanded roles on study teams as Co-Investigators	Understanding of research, processes, ethics and outcomes, as well as better understanding of rights and protections. More knowledge about leadership role and responsibilities and how to run or participate in a board.
Interpersonal	Participant/patients' relatives	Some shared family benefit from compensation, stipend, vouchers	opportunity to get useful health information, access to health care and health providers, possibility for early diagnosis if relatives is found to be predisposed to any disease. development of personalised medicine						Understanding of research, processes, ethics and outcomes, as well as better understanding of rights and protections,
Individual	Research participants	Compensation through direct monetary remuneration for participation, stipends, vouchers e.g. vouchers for groceries, or airtime/mobile phone credits.	Access to health care and health informations, early diagnosis of disease, feedback of future incidental findings from secondary data use.	Access to a more advanced treatment or diagnostic tools through research programmes.	It may be possible for participants to keep research equipment e.g. mobile phones, wearable tech.				Information provided to inform making better choices. Understanding of research, processes, ethics and outcomes, as well as better understanding of rights and legal protections.
Individual	Specialist service providers in the private sector	Compensation, stipend, vouchers	Improved health care (using outputs from research); opportunities for precision medicine	Improved infrastructure at local health care facilities (e.g. new fridge), improved health care experience; access to health care services provided through research participation that may otherwise be unavailable/unaccessible	In some programmes it may be possible to assist patients with specialised equipment to help with their health condition e.g. orthotics, wearable tech, mobile phones for health apps	Training received in specialist areas to assist with participating in research programmes	Skills and knowledge to offer more services to clients e.g. Able to recommend new genetic tests for diseases	opportunity to become member of patient advocacy group, opportunity for scholarship/funding for project/academic advancement	Education of patients regarding new information available through research, better understanding of their health condition and prevention or treatment options.
Individual	Specialist service providers in the public sector	New research-related funding opportunities become available.		Access to better infrastructure to support perform functions.	Access to better/highly specialised equipment.	Taught new skills required to conduct research, provided up-to-date information on recent developments in public health and clinical approaches.	Improved efficiency and scope of services the provider may propose, e.g. wider scope of tests or medications that might be offered.	Recognition of contributions in published output.	Mentoring, enhancing institutional knowledge through interactions with other professionals in the research network.
Individual	health care providers	Salaries may be augmented with salary contributions for funded research activities.	Improved service delivery achieved through improved knowledge.	Improved work enviroment and maybe even improved safety for things such as infection control	new equipment could improve workload (e.g. automated DNA extraction machines), efficiency, accuracy and safety	Taught new skills required to conduct research; provided up-to-date information about new diagnostic and treatment approaches.	New skills and learnings might lead to a wider scope of services, diagnostic and treatment applications when serving clients	Participation in research programmes may boost CV when applying for employment opportunities	Access to upto date data from their patients which can help them to recognise both population wide and individual level trends. This can help them provide better care or even be prepared for any future threats to their patients e.g. People living with diabetes, people living with HIV
Individual	Researchers - PIs and senior	Salary; promotion/tenure opportunites; grant funding for research and salary support; commercialization which may result in personal financial gains through inventions, commercialization of products, or patents. Indirect funds (percentage of) from grant awards can be contributed to the investigator's professional financial account within their institution to support additional training, conference attendance, and equipment beyond the scope of the study.	Healthcare coverage provided via indirects of grants offered through institutions.	Improved infrastructure and facilities for more research; individual access to institutional resources through receipt of grant funding, e.g. post award resources and services available to funded investigators.	Laptops and personal equipment provided through grants; access to specialised research equipment to enhance research activities.	Opportunities to attend workshops and conferences to broaden knowledge. Development of skill expertise through the conduct of studies.	Researchers may be able to offer a wider array of research and analysis skills for future collaborations or to support research colleagues and supervise students.	Specialist and professional skills development. Academic Promotion. Access to wider networks of researchers and funders; greater impact to the field. Opportunities for awards and recognition within institutions, professional organizations, and scientific communities.	Expanded knowledge of research methods and scientific areas studied through conducting studies.
Individual	Researchers - Junior	Stipends/salary support.	Healthcare coverage offered through institutions, funded via indirect overheads from grants.	Well-resourced research infrastructure and environment in which to gain experience, provides a well-resourced learning environment.	Laptops and personal equipment provided through grants. Access to specialised research equipment to enhance research activities.	Building research and dissemination skills. Sufficient mentors are available due to research funding.	Junior researchers may gain training and experience that provide skills in a future services-based career.	Specialist and professional skills development. Access to wider networks of researchers and funders; Opportunities to expand CVs/biosketches through research experience.	Learning from being mentored, and gaining insights to institutional knowledge. Hands-on research opportunities and the opportunity to learn from team and cross disciplinary collaboratives.
Individual	Postgrad students, postdocs	Tuition remuneration; stipends; coverage for conference attendance/presentation costs.	Healthcare coverage offered through institutions, funded via indirect overheads from grants.	Well-resourced research infrastructure and environment in which to gain experience, provides a well-resourced learning environment.	Laptops and personal equipment provided through grants. Access to specialised research equipment to enhance research activities.	Building skills in managing and analysing data (e.g.assisting with data analysis in a health crisis)	More skills and training may provide more well-equipped entry-level professionals for the health service	Specialist and professional skills development. Access to onward training and employment through wider research networks	Learning from being mentored, and gaining insights to institutional knowledge. Learning how to acquire, filter and use new knowledge.
Individual	General population (not involved in the research projects directly)		Flow through effects where research engagement generally improves employment opportunities, economic activity, education and scientific citizenship in an individual's community or district, e.g. downstream positivie effects of hosting a large population cohort study in a particular community or district, as experienced by individuals in that community.	Access to improved infrastructure and facilities for general use, or healthcare related activities.	Benefit from equipment that have multiple uses in the provision of services or health care e.g. the GeneXpert machine which is mainly use to rapidly diagnose TB and point of care, can also be used to measure HIV viral load.	Upskilling of individuals interacting with the research environment, e.g. Community Advisory Board (CAB) members, as well as more general dissemination of information through brochures, online media and other publicly-available resources.	Benefiting from access to services that have been increased through research activity, e.g. ramped up HIV/TB testing in the community.	Upskilling of individuals e.g. Community Advisory Board (CAB) members.	Developing a broader understanding of health conditions and the research process through appropriate level public communciations, media and engagement about research programmes and their findings.
